# High-Throughput CAMP Assay (HiTCA): A Novel Tool for Evaluating the Vitamin D-Dependent Antimicrobial Response

**DOI:** 10.3390/nu15061380

**Published:** 2023-03-13

**Authors:** Carter Gottlieb, Mason Henrich, Philip T. Liu, Vahe Yacoubian, Jeffery Wang, Rene Chun, John S. Adams

**Affiliations:** 1 Department of Orthopaedic Surgery, University of California, Los Angeles, CA 90095, USA; 2 Department of Molecular, Cell & Developmental Biology, University of California, Los Angeles, CA 90095, USA; 3 Department of Microbiology, Immunology and Molecular Genetics, University of California, Los Angeles, CA 90095, USA

**Keywords:** cathelicidin, CAMP, LL-37, vitamin D, calcitriol, antimicrobial, immunity, innate immunity

## Abstract

Vitamin D is known to modulate human immune responses, and vitamin D deficiency is associated with increased susceptibility to infection. However, what constitutes sufficient levels or whether vitamin D is useful as an adjuvant therapeutic is debated, much in part because of inadequate elucidation of mechanisms underlying vitamin D’s immune modulatory function. Cathelicidin antimicrobial peptide (CAMP) has potent broad-spectrum activity, and the *CAMP* gene is regulated in human innate immune cells by active 1,25(OH)_2_D_3_, a product of hydroxylation of inactive 25(OH)D_3_ by CYP27B1-hydroxylase. We developed a CRISPR/Cas9-edited human monocyte-macrophage cell line containing the *mCherry* fluorescent reporter gene at the 3′ end of the endogenous *CAMP* gene. The High Throughput CAMP Assay (HiTCA) developed here is a novel tool for evaluating CAMP expression in a stable cell line that is scalable for a high-throughput workflow. Application of HiTCA to serum samples from a small number of human donors (n = 10) showed individual differences in CAMP induction that were not fully accounted for by the serum vitamin D metabolite status of the host. As such, HiTCA may be a useful tool that can advance our understanding of the human vitamin D-dependent antimicrobial response, which is being increasingly appreciated for its complexity.

## 1. Introduction

Cathelicidins are cationic antimicrobial peptides with potent broad-spectrum activity targeting gram-positive and gram-negative bacteria (e.g., *Escherichia coli*, *Mycobacterium leprae*, *Mycobacterium tuberculosis*, and *Staphylococcus aureus*), fungi (e.g., *Candida albicans* and *Aspergillus* spp.), and viral pathogens (e.g., HIV-1 and SARS-CoV-2) [1,2]. The mechanisms by which cathelicidins act on microbes are diverse, including microbial cell membrane disruption [3,4], inhibition of microbial replication [5], and recruitment and regulation of the host’s innate and adaptive immune system [6]. The human cathelicidin antimicrobial peptide gene (*CAMP*), which is highly expressed by innate immune cells such as monocytes, macrophages, dendritic cells, and neutrophils encountering microbial products, encodes the CAMP precursor protein that undergoes proteolytic cleavage to produce the single antimicrobial peptide, LL-37 [7]. The expression of antimicrobial peptides is regulated by various macro- (e.g., branched-chain amino acids, short-chain fatty acids, and lactose) and micro-nutrients (e.g., vitamin D, vitamin A, and zinc) [8]. Among these, vitamin D is critical for CAMP expression. Owing to the insertion of a retrotransposon bearing a consensus vitamin D response element (VDRE) in the proximal *CAMP* promoter, *CAMP* is transcriptionally responsive to the active vitamin D metabolite, 1,25-dihydroxyvitamin D (either in the form of 1,25(OH)_2_D_3_ or 1,25(OH)_2_D_2_) bound to the vitamin D receptor (VDR) in humans and higher non-human primates [9]. Insufficient provision of prohormone 25-hydroxyvitamin D (either in the form of 25(OH)D_3_ or 25(OH)D_2_) and/or inadequate intracellular conversion of substrate 25(OH)D to product 1,25(OH)_2_D by the CYP27B1-hydroxylase in microbe-activated monocyte-macrophages can lead to a deficiency in CAMP production and secretion [10]. 

The ability of vitamin D metabolites to modulate *CAMP* expression may explain the role of vitamin D in innate and adaptive immunity [11,12,13]. Deficient vitamin D status of the human host, manifested by low circulating levels of 25(OH)D, has historically been associated with increased rates of influenza [14], viral upper respiratory tract infection [15], pneumonia [16], bacterial vaginosis [17], HIV [18,19], and active *Mycobacterium tuberculosis* [20], among other microbial infections and disease states [21]. More recently, vitamin D deficiency has been associated with increased SARS-CoV-2 infection risk [22,23], greater COVID-19 severity [24], and delayed COVID-19 recovery [25] though some studies did not find an association [26,27], leaving the role of vitamin D as an immune modulator in COVID-19 debated.

The earliest attempt to reduce the incidence and/or severity of microbial infection with vitamin D dates to the early twentieth century when sunlight exposure was used to treat patients with *M. tuberculosis* [28]. Despite these early findings, the administration of vitamin D and its metabolites in randomized controlled trials of patients with infectious and/or inflammatory diseases was not pursued until almost a century later. The results of these more recent trials have been mixed, although some trials with “negative” results failed to give an adequate dose of vitamin D to significantly raise serum levels of 25(OH)D [29]. The strongest evidence of a vitamin D antimicrobial effect arose from effective supplementation studies in the context of influenza [30] and upper respiratory tract infections [31,32]. Again, since the onset of the COVID-19 pandemic, vitamin D has become a topic of debate, as some supplementation studies, although variable, have shown a reduced requirement for ICU treatment among patients hospitalized with COVID-19 [33] and accelerated recovery from pulmonary symptoms in non-hospitalized SARS-CoV-2-infected patients [34,35]. 

Our laboratory has previously developed an ex vivo *CAMP* expression assay, whereby primary cultures of VDR-expressing, single donor monocyte-macrophages are conditioned with heterologous donor serum before measuring *CAMP* expression by qPCR [36]. The assay has proved valuable for (i) screening of donor serum to alter ex vivo *CAMP* expression based on the concentration of 25(OH)D in the conditioning serum [37], (ii) identifying demographic differences in the ability of human serum to induce *CAMP* expression [10], (iii) elucidating a mechanism by which *Mycobacterium leprae* evades the host immune response [38], and (iv) observing greater *CAMP* induction by serum from young adults with HIV after intervention with oral vitamin D_3_ [39].

This ex vivo *CAMP* assay has been an essential tool to study the vitamin D-mediated immune response in activated primary cultures of human monocyte-macrophages. However, the assay suffers from a few key weaknesses. (1) *Donor variability*. The ex vivo assay requires both a variable source of monocyte-macrophages and heterologous human serum. Because the monocyte-macrophages come from a single donation, there is a limit to the number of cells that can be acquired, and long-term storage of primary monocyte-macrophages is not feasible. Therefore, every experiment, although internally consistent, has variability between itself and other experiments, which use cultured monocyte-macrophages from a different donor. Because single donor primary monocyte-macrophages are used in each experiment, the traditional assay is not ideal for many applications, such as in a setting where the readout of the assay might be compared to a standard cohort. (2) *Low throughput*. The ex vivo assay requires extensive sample handling to prepare peripheral blood mononuclear cells (PBMCs) from whole blood, select for plastic-adherent cells, incubate with vitamin D metabolites in serum-conditioned media, extract RNA, synthesize cDNA by reverse transcription, and perform qPCR. The sample handling time makes the assay difficult to scale beyond a single 96-well plate and introduces opportunities for user error and sample degradation. (3) *Contamination*. Using plastic adherence as a method to purify monocytes leads to contamination with a small number of neutrophils, lymphocytes, or other *CAMP*-expressing, plastic-adherent cells that may interfere with the assay [40,41]. 

**Figure 1:**
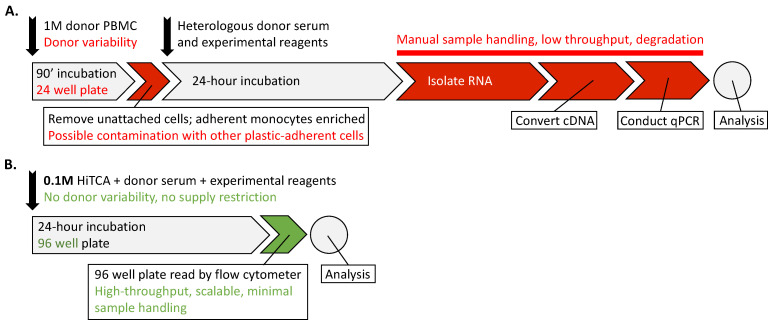
**HiTCA enables high-throughput, scalable, and reliable bioassay of CAMP.** A workflow comparison between (**A**) the ex vivo *CAMP* assay, which employs 1 million (1 M) PBMC, and (**B**) the High Throughput CAMP Assay (HiTCA), which employs 100,000 (0.1 M) HiTCA cells. Difficulties of the ex vivo assay are written in red text. The means by which HiTCA addresses these issues are written in green text.

We circumvented these three problems by creating a CRISPR/Cas9-directed knock-in of a cDNA containing the *mCherry* fluorescent reporter gene at the 3′ end of the endogenous *CAMP* gene in the human CYP27B1- and VDR-expressing SC monocytic cell line (ATCC, CRL9855). This High Throughput CAMP Assay (HiTCA) immortalized cell line can be propagated indefinitely to yield consistent results in a scalable high-throughput workflow. It requires minimal sample handling, and it uses a homogenous source of target monocyte-macrophages with no contamination by other immune cells found in plastic-adherent monocyte-macrophages preparations (Figure 1).

## 2. Materials and Methods

### 2.1. Insertion of mCherry at 3′ End of CAMP Gene in Human SC Cells

CRISPR/Cas9-mediated knock-in of *mCherry* (~711 bp) at the 3′ end of the *CAMP* gene (Ensembl Gene ID: ENSG00000164047; Ensembl Transcript ID: ENST00000296435; NCBI Ref: NM_004345) in the human monocyte-macrophage SC cell line (ATCC, CRL9855) was performed by Synthego Corporation (Redwood City, CA, USA). To generate homozygous *CAMP^mCherry^* human SC cell clones, ribonucleoproteins containing the Cas9 protein and a synthetic chemically modified sgRNA (CACACACTAGGACTCTGTCC) targeting the 3′ end of the human *CAMP* gene (chr3: *CAMP*-48225414) were produced at Synthego. In addition, donor plasmids containing *mCherry* flanked by homologous arms to the target region were produced at Synthego. 

**Figure 2:**
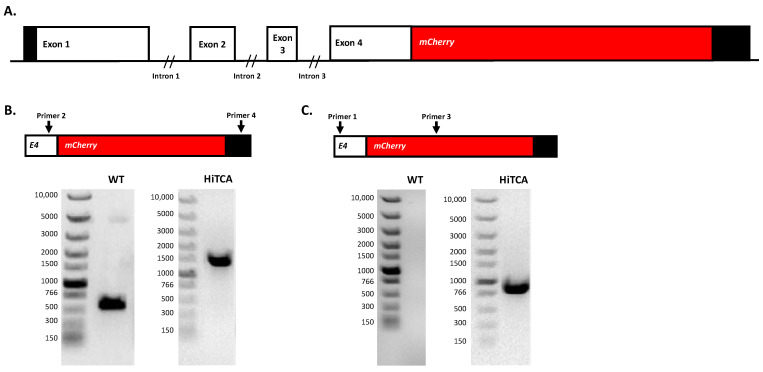
**Design and validation of homozygous *CAMP^mCherry^* human SC cell line.** (**A**) Schematic representation of *mCherry* insertion at the 3′ end of the *CAMP* gene in the human SC cell line using CRISPR/Cas9. Successful knock-in of *mCherry* in HiTCA cells but not in wild type (WT) SC cells was confirmed by PCR genotype analysis of (**B**) full-length insert (PCR product of primer 2 (forward) and primer 4 (reverse); expected size of 1348 bp in HiTCA cells and 529 bp in WT) and (**C**) insert junction (PCR product of primer 1 (forward) and primer 3 (reverse); expected size of 851 bp in HiTCA cells and 0 bp in WT).

Together, Cas9-sgRNA complexes and donor plasmids were electroporated into SC cells using Synthego’s optimized protocol. Editing efficiency was assessed upon recovery 48 h following electroporation. Genomic DNA was extracted from a portion of the cells, PCR amplified and sequenced using Sanger sequencing. The sequencing results were processed using Synthego Inference of CRISPR Edits software (ICE, ice.synthego.com). Edited cell pools identified by ICE were seeded at <1 cell/well using a single-cell printer into 96- or 384-well plates to create monoclonal cell populations. All wells were imaged every 3 days to ensure expansion from a single-cell clone. Ultimately, a monoclonal population of homozygous *CAMP^mCherry^* human SC cells (referred to hereafter as HiTCA cells) was identified and confirmed by PCR genotyping (Figure 2). The primers used for PCR genotype analysis were as follows: primer 1, 5′-AAGGTGAGTGGGCTGTTCTG-3′; primer 2, 5′-ATTGGGTGTAGGGATTCAATCACA-3′; primer 3, 5′-AAGCGCATGAACTCCTTGAT-3′, primer 4, 5′-GAGTAGAGCTCCTTAATTCCCCAC-3′.

### 2.2. Cell Culture and Maintenance

For maintenance, HiTCA and non-gene edited wild type (WT) SC cells were cultured in media composed of 88% Gibco RPMI (Thermo Fisher Scientific, Waltham, MA, USA), 10% FBS, 1% Sodium Pyruvate (100 mM), and 1% Pen-Strep antibiotic. The cell cultures were propagated in 25 cm^2^ canted neck cell flasks with vented caps at a density between 2.0 × 10^6^ and 2.0 × 10^7^ cells/mL. The flasks were incubated at 37 °C and 5% CO_2_. While maintaining the number of cells in culture, they were passaged every 2–3 days by decanting half of the culture media and adding an equal volume of fresh culture media. Cells were tested periodically for viability with trypan blue and counted with a Bio-Rad TC20 Cell Counter (Bio-Rad Laboratories, Hercules, CA, USA) to ensure viability over 90%. 

### 2.3. HiTCA Protocol

**Figure 3:**
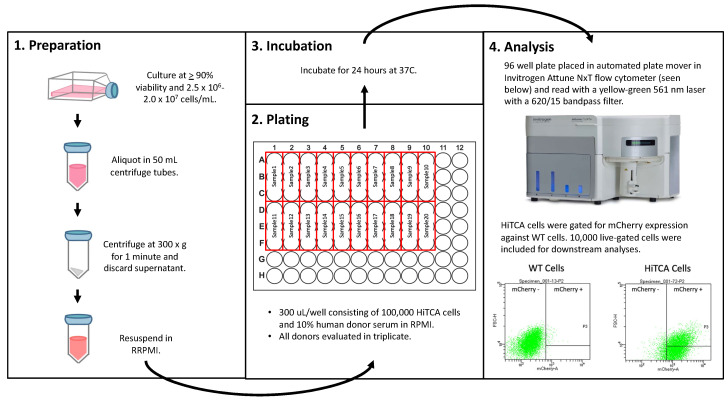
**HiTCA Protocol.** HiTCA protocol details include (1) preparation, (2) plating, (3) incubation, and (4) analysis.

For high-throughput analyses, HiTCA cells were added to 50 mL conical tubes and centrifuged at 300× *g* for 10 min. The media was removed, and the pellet was resuspended in an equal volume of RPMI. Cells were then plated in a 96-well plate at a concentration of 100,000 cells/well. Depending on the specifics of the experimental conditions, human serum along with treatment substrates (e.g., ethanol and vitamin D metabolites) were added to each well such that the total volume/well was 300 uL. Each sample was assayed in triplicate. The cells were then incubated for 24 h at 37 °C. While present in the culture media used for cell maintenance, FBS, sodium pyruvate, and Penstrep antibiotic were all absent from culture media used for incubation and analysis. After the 24 h incubation period, high-throughput flow cytometry analysis was performed on an Invitrogen Attune NxT (Thermo Fisher Scientific). The samples were read with a yellow–green 561 nm laser with a 620/15 bandpass filter. All cells were gated to exclude non-viable cells and doublets. WT cells acted as negative controls for CAMP^mCherry^ gating, with gating parameters set such that WT cells were 1% CAMP^mCherry^ positive. Ultimately, 10,000 live cells in single-cell suspension were included for data collection using Invitrogen Attune NxT built-in software (Thermo Fisher Scientific) and analysis using FlowJo. GraphPad Prism 8 was used for statistical analyses and figure production. This protocol is summarized in Figure 3.

### 2.4. Induction of CAMP^mCherry^ in Human SC Cell Clones by 25(OH)D_3_ and 1,25(OH)_2_D_3_

We evaluated responsiveness of HiTCA cells to 25(OH)D_3_ via endogenous CYP27B1-hydroxylase catalysis of inactive precursor 25(OH)D_3_ to active 1,25(OH)_2_D_3_. HiTCA protocol was followed as described in Figure 3. During the incubation phase, HiTCA cells were incubated with escalating concentrations of 25(OH)D_3_ (1 nM, 10 nM, 100 nM). EtOH and 10 nM 1,25(OH)_2_D_3_ treatments were included as negative and positive controls.

### 2.5. Application of Pooled Human Serum to HiTCA

We assessed the influence of human serum on the CAMP response using HiTCA. We followed HiTCA protocol as described in Figure 3 and maintained the incubation conditions described in Section 2.4 while also incorporating increasing concentrations (0%, 0.3%, 3%, 10%) of pooled human serum obtained from Omega Scientific (Westlake Village, CA, USA).

### 2.6. Application of Donor-Specific Serum to HiTCA

We tested HiTCA with a small number of single human donor serum samples. Specifically, we conducted a pilot experiment with serum from 5 White males and 5 Black males obtained from Innovative Research (Novi, MI, USA). The whole blood was collected from donors in an FDA-approved collection center. Each unit was tested for the standard FDA-required viral markers, and found negative for HBsAg, HCV, HIV-1, HIV-2, HIV-1Ag or HIV-1 NAT, ALT, and syphilis using FDA-approved methods. Demographic information provided by Innovative Research for each donor (race, sex, and age) is summarized in Table 1. Human serum samples were measured for 25(OH)D and 1,25(OH)_2_D by LC-MS/MS (Waters, Manchester, UK) in the laboratory of Martin Hewison (Institute of Metabolism and Systems Research, the University of Birmingham, Birmingham B15 2TT, UK) using methods described in [42] using guidelines established by the US Food and Drug Administration guidelines for analysis of vitamin D metabolites [43].

HiTCA protocol was followed as described in Figure 3. During the incubation phase, HiTCA cells were incubated with donor specific serum (n = 10) at variable serum concentrations (0.3%, 10%) and exogenous vitamin D metabolite treatments (EtOH, 100 nM 25(OH)D_3_, 10 nM 1,25(OH)_2_D_3_). 

## 3. Results

### 3.1. Endogenous 25(OH)D_3_-to-1,25(OH)_2_D_3_ Activation in HiTCA Cells

**Figure 4:**
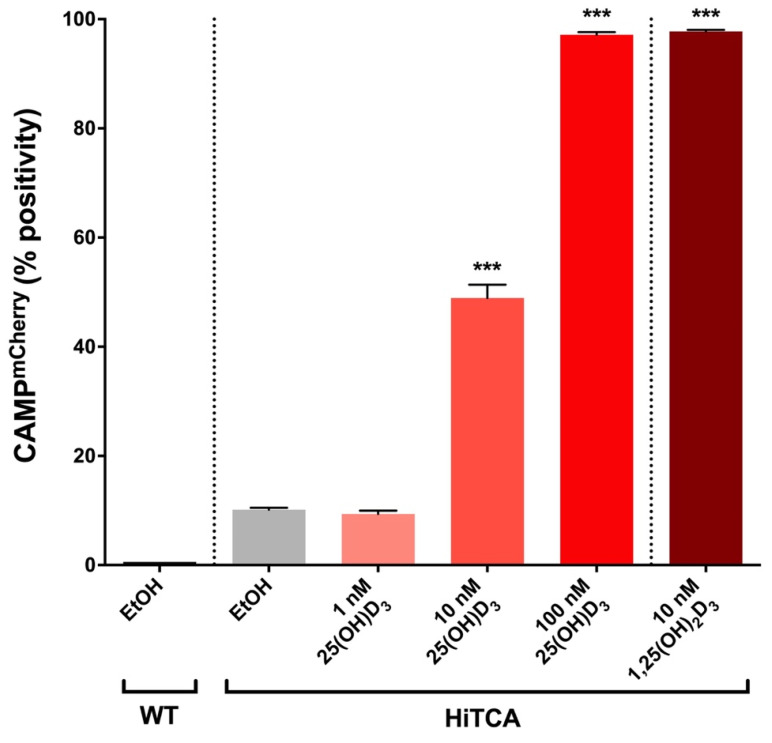
**25(OH)D_3_ and 1,25(OH)_2_D_3_ induce CAMP^mCherry^ response in HiTCA cells.** HiTCA cells were incubated in RPMI, and either EtOH (equal volume as vitamin D metabolite solvent) or vitamin D metabolites (1 nM 25(OH)D_3_, 10 nM 25(OH)D_3_, 100 nM 25(OH)D_3_, 10 nM 1,25(OH)_2_D_3_) at 37 °C for 24 h prior to flow cytometry analysis. EtOH-treated WT cells were gated to be 1% CAMP^mCherry^ positive. Therefore, all non-WT columns represent the percentage of 10,000 live-gated single-cells that express CAMP^mCherry^ above the WT control levels. Each column represents n = 3 biological replicates ± SD. Asterisks represent significant differences (*** *p* < 0.001) between vitamin D metabolite-treated HiTCA cells and their EtOH-treated equivalent.

HiTCA cells demonstrated a dose-dependent response to 25(OH)D_3_ (Figure 4), as EtOH-treated and 1 nM 25(OH)D_3_-treated cells exhibited baseline expression (10% CAMP^mCherry^ positive) that increased to 50% in response to 10 nM 25(OH)D_3_ and nearly 100% in the presence of 100 nM 25(OH)D_3_, comparable to levels observed in 10 nM 1,25(OH)_2_D_3_-treated cells. These results strongly suggest that this *CAMP^mCherry^* human SC cell line is capable of converting 25(OH)D_3_ to its transcriptionally active form, 1,25(OH)_2_D_3,_ via endogenously expressed CYP27B1-hydroxylase.

### 3.2. Higher Human Serum Concentration Attenuates the HiTCA Response

**Figure 5:**
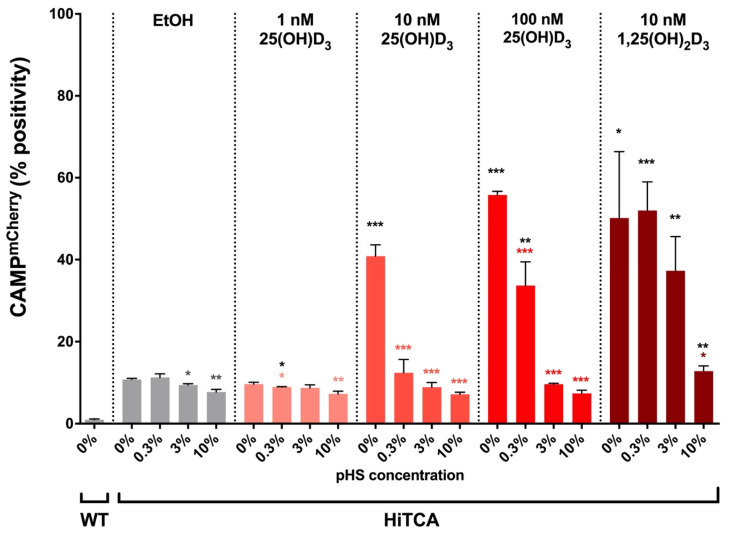
**Pooled human serum dose-dependent attenuation of HiTCA CAMP^mCherry^ response.** HiTCA cells were incubated in RPMI, EtOH (equal volume as vitamin D metabolite solvent), vitamin D metabolites (1 nM 25(OH)D_3_, 10 nM 25(OH)D_3_, 100 nM 25(OH)D_3_, 10 nM 1,25(OH)_2_D_3_), and pooled human serum (pHS; 0%, 0.3%, 3%, 10%) at 37 °C for 24 h prior to flow cytometry analysis. EtOH-treated WT cells were gated to be 1% CAMP^mCherry^ positive. Therefore, all non-WT columns represent the percentage of 10,000 live-gated single-cells that express CAMP^mCherry^ above the WT control levels. Each column represents n = 3 biological replicates ± SD. Black asterisks represent significant differences (* *p* < 0.05, ** *p* < 0.01, *** *p* < 0.001) between vitamin D metabolite-treated HiTCA cells and their EtOH equivalent with the same pHS exposure. Colored asterisks represent significant differences (* *p* < 0.05, ** *p* < 0.01, *** *p* < 0.001) between 0% pHS-treated HiTCA cells and non-zero pHS-treated HiTCA cells of the same vitamin D metabolite treatment group.

Coincubation of HiTCA cells with pooled human serum resulted in a dose-dependent attenuation of CAMP^mCherry^ expression as the pooled human serum concentration was increased from 0% to 10% (Figure 5). The response of HiTCA cells to serum was consistent across all vitamin D metabolite treatment conditions.

### 3.3. Donor Specific Serum Yielded Variable Responses

**Figure 6:**
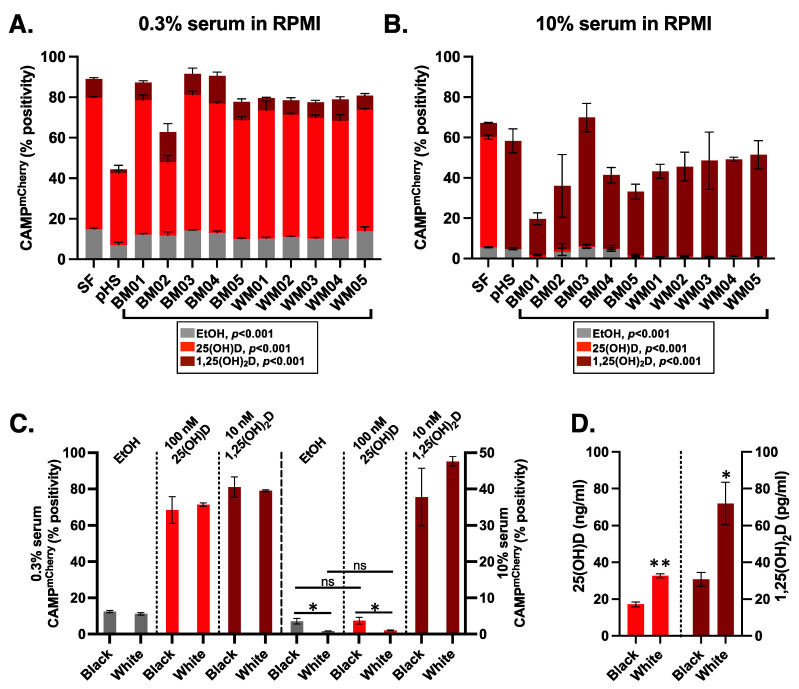
**HiTCA response to varying human donor serum concentrations.** HiTCA cells were treated with either EtOH (equal volume as vitamin D metabolite solvent), 100 nM 25(OH)D_3_, or 10 nM 1,25(OH)_2_D_3_ and one of the following serum treatments: serum-free (SF: no exogenous serum added), pooled human serum (pHS), or individual donor serum. Individual donor serum concentration and culture media conditions were varied as follows: (**A**) 0.3% serum in RPMI and (**B**) 10% serum in RPMI. Data represent the percentage of 10,000 live-gated single-cells that express CAMP^mCherry^ with gate of EtOH-treated WT cells set to 1% positive. Each column represents n = 3 biological replicates ± SEM. Columns representing the three vitamin D metabolite treatments are superimposed according to serum treatment. Vitamin D metabolite treatments are color-coded according to the figure legend. Statistical significance *p* < 0.001 was attained between individual donors in all treatment conditions by ANOVA. (**C**) Data from panels A and B are displayed as comparisons of average percentage CAMP^mCherry^ signals from the Black (n = 5) and White (n = 5) samples in the various indicated test conditions (y-axis, 0.3% serum on left and 10% serum on right) and above the column graphs (EtOH, 100 nM 25(OH)D_3_, or 10 nM 1,25(OH)_2_D_3_). Differences between Black and White samples were observed in only two conditions, * *p* < 0.05. Differences between EtOH and 100 nM 25(OH)D_3_, and EtOH and 10 nM 1,25(OH)_2_D_3_ within corresponding Black/White groups were all significant (*p* < 0.01) with the exception of two indicated non-significant (ns) comparisons. (**D**) Measured serum 25(OH)D (left axis) and 1,25(OH)_2_D (right axis) mean concentrations of the Black and White samples used in study. * *p* < 0.05 and ** *p* < 0.001 was observed comparing Black with White samples.

We then tested HiTCA with a small number of single human donor serum samples. Specifically, we conducted a pilot experiment with serum from five White males and five Black males (Figure 6, Table 1). Based on the results of our pooled human serum experiments (Figure 5), we decided to compare 0.3% (Figure 6A) and 10% (Figure 6B) human donor serum to determine whether differential CAMP expression could be observed across individual donors. In both serum settings (Figure 6A,B), we identified statistically significant differences (*p* < 0.001) in CAMP^mCherry^ expression across individual donors when vehicle incubated (EtOH; gray bars) and when co-incubated with exogenous 100 nM 25(OH)D_3_ (light red bars) or 10 nM 1,25(OH)_2_D_3_ (dark red bars). Consistent with our previous result (Figure 5), we observed attenuation of the HiTCA CAMP^mCherry^ 25(OH)D_3_ response in the presence of high serum concentrations (10%; Figure 6B) as compared to low serum concentration (0.3%; Figure 6A). In 0.3% serum, CAMP^mCherry^ response was elevated (5 to 7-fold, *p* < 0.001) above EtOH controls when co-incubated with 100 nM 25(OH)D_3_ and 10 nM 1,25(OH)_2_D_3_ (Figure 6C, left). In 10% serum, differences between EtOH and 100 nM 25(OH)D_3_ were not significant, while CAMP^mCherry^ response was elevated (10 to 54-fold, *p* < 0.01) above EtOH controls when co-incubated with 10 nM 1,25(OH)_2_D_3_ (Figure 6C, right). In the six conditions where White and Black samples could be compared, only in two conditions (10% serum, EtOH and 100 nM 25(OH)D_3_ added) was the CAMP^mCherry^ response statistically different between Black and White samples (Figure 6C). Unexpectedly, the Black samples had higher CAMP^mCherry^ responses in those conditions (4-fold higher in 10% serum EtOH, *p* < 0.05; 3.5-fold higher in 10% serum 100 nM 25(OH)D_3_, *p* < 0.05) compared to White samples. This is surprising given that the serum from Black samples was lower than White samples in both their mean 25(OH)D (17.14 ng/mL, SD = 2.93 vs. 32.72 ng/mL, SD = 2.40; *p* < 0.001) and 1,25(OH)_2_D (30.74 pg/mL, SD = 8.34 vs. 71.97 pg/mL, SD = 25.62; *p* < 0.05) levels (Figure 6D).

## 4. Discussion

Investigations of the vitamin D-dependent antimicrobial response require time-consuming methods for characterizing *CAMP* and/or CAMP expression, including the ex vivo *CAMP* assay [36]. The ex vivo *CAMP* assay designed previously was limited by potentially confounding issues of donor variability in primary cultures of human monocyte-macrophages, variability in the expression of CYP27B1-hydroxalase and VDR in that cell, low-throughput capacity, contamination with other *CAMP*-expressing cells, and readout at the level of mRNA not protein. The High Throughput CAMP Assay (HiTCA) addresses each of the limitations of the ex vivo *CAMP* assay by employing a homogenous source of *CAMP^mCherry^* monocyte-macrophages expressing fluorescently-labeled CAMP protein in a fully scalable, high-throughput workflow that requires minimal manual sample handling. 

CAMP^mCherry^ response was strongly induced as expected when exogenous 25(OH)D_3_ and 1,25(OH)_2_D_3_ were added to HiTCA cultured without serum (Figure 4). However, the addition of human serum to HiTCA strongly and consistently attenuated CAMP^mCherry^ response. Specifically, HiTCA showed CAMP^mCherry^ response to 100 nM 25(OH)D_3_ and 10 nM 1,25(OH)_2_D_3_ in 0.3% serum-supplemented media (Figure 5 and Figure 6), while only 10 nM 1,25(OH)_2_D_3_ induced CAMP^mCherry^ in 10% serum-supplemented media (Figure 5 and Figure 6). These differences are likely due to vitamin D binding protein (DBP) that has a very high affinity for 25(OH)D_3_ and much lower affinity for 1,25(OH)_2_D_3_, resulting in the squelched response to 25(OH)D_3_ in the 10% serum setting. 

Potentially interesting are the differences seen among individual donor human serums and the difference in response of pooled human serum compared to the individual donor serums. Large volumes of pooled human serum prepared by commercial vendors may have significantly different sample handling procedures that may yield differences when compared against small-volume individual donor serum preparations commonly found in studies analyzing clinical samples or purchased from vendors. Perhaps, commercial high-volume serum preparation differentially alters some serum components that support/suppress the CAMP^mCherry^ response.

As for the inter-donor variability, significant racial differences in both 25(OH)D and 1,25(OH)_2_D levels have been described in the past [44]. In our samples, the Black serum samples were significantly lower in their 25(OH)D and 1,25(OH)_2_D levels compared to the White serum samples (Figure 6D). Yet, in two of the assay conditions, 10% serum EtOH and 10% serum 100 nM 25(OH)D_3_, the CAMP^mCherry^ response was significantly higher in the Black samples (Figure 6C). While further investigation is required to support this claim with certainty, these results suggest that regulation of the CAMP^mCherry^ response is more complicated than just the measurable serum 25(OH)D or 1,25(OH)_2_D levels. It is possible that some serum components that support/suppress the CAMP^mCherry^ response differ among individuals, especially among individuals of different ethnicities. Some have proposed that genetic polymorphisms in the serum vitamin D binding protein (DBP) could account for differences in vitamin D-dependent processes through differences in the affinity of DBP for vitamin D metabolites among the polymorphisms [45,46]. However, biochemical studies of DBP affinity and measurements of free 25(OH)D among subjects with different DBP polymorphisms indicate that affinity differences are small [47,48,49]. Other proposed regulators of CAMP induction are cytokines (e.g., IL-15, IL-4, and IFN-γ), which can vary both qualitatively and quantitatively across individual donors and are known to impact vitamin D responsiveness in cells [50,51,52]. Further, microRNAs may act as regulatory molecules, perhaps through factors that stimulate/suppress microRNA expression in monocyte-macrophages [53] or exosome-delivered microRNAs that affect vitamin D response [54]. With consideration of the complexity of this process, Calberg and Haq recently conceptualized the vitamin D response index [55], which suggests that individual differences in diverse biochemical and clinical parameters, and not vitamin D status alone, govern response to vitamin D and requirement for vitamin D supplementation. In addition, there may be vitamin D-independent mechanisms at play, including differences in serum levels of other macro- (e.g., branched-chain amino acids, short-chain fatty acids, and lactose) and micro-nutrients (e.g., vitamin D, vitamin A, and zinc) known to regulate antimicrobial peptide expression [8]. As such, HiTCA is a proof-of-concept of the type of novel resource needed in the field to facilitate the analysis of large numbers of samples to explore the intricacies of the CAMP response.

Despite the discovery potential, we are aware of various limitations of our method and analysis presented in this report. First, our experiments employed a moderate sample size (n = 10; Figure 6). Second, our experiments were conducted using sera from young (20 to 40-year-old) male subjects (Table 1) and, therefore, did not test a fuller possible range of individual differences that the inclusion of various ages and genders might provide. Third, although the race, sex, and age of the donors were known, we had no further knowledge of the donors’ physical characteristics, such as inflammatory state, past medical history, drug or supplement use, and serum levels of other CAMP-influencing nutrients. As such, while our current work serves as a proof-of-concept for the HiTCA assay, in the future, we plan to apply HiTCA to a larger sample with diverse demographic (race, sex, age, etc.) and physical characteristics. In addition, we plan to apply HiTCA in the context of various pathologic perturbations, including exposure to infection and/or inflammatory mediators. Finally, the somewhat muted response to 25(OH)D in 10% serum settings could indicate that the cell line has modest CYP27B1 expression, which could be addressed in future iterations of HiTCA. However, our observation of individual differences in CAMP^mCherry^ induction is consistent with the known challenges of dissecting the individualized parameters that drive immune response. 

## 5. Conclusions

We developed the High Throughput CAMP Assay (HiTCA), a novel tool for evaluating CAMP expression in a stable, *CAMP^mCherry^*-expressing, monocyte-macrophage innate immune cell line. HiTCA is sensitive to individual differences in CAMP induction and yields consistent results in a fully scalable, high-throughput workflow. This proof-of-principle study yielded findings consistent with evidence from both the biological and clinical realms that support individual diversity in the complex vitamin D-dependent antimicrobial processes. HiTCA, further refined and tested in a larger number of samples, may prove to be a valuable means of identifying novel, serum-based factors that impact the immune regulatory function of vitamin D. With this knowledge, the field may more adequately assess and apply vitamin D as a sole or adjuvant therapeutic for infectious diseases. 

## Figures and Tables

**Table 1:** Demographic and vitamin D metabolite data of donor specific serum.

Sample	Race	Sex	Age	25(OH)D (ng/mL)	1,25D (pg/mL)
BM01	Black	Male	26	18.45	35.66
BM02	Black	Male	38	21.05	35.66
BM03	Black	Male	38	18.68	40.09
BM04	Black	Male	37	14.38	24.96
BM05	Black	Male	26	13.13	17.39
WM01	White	Male	29	31.92	76.75
WM02	White	Male	24	29.15	67.58
WM03	White	Male	38	34.29	107.15
WM04	White	Male	39	36.24	80.32
WM05	White	Male	27	32.02	28.02

## Data Availability

All data generated or analyzed during this study are included in this published article.
